# Structural characterization and active site prediction of keratinase from Bacillus flexus

**DOI:** 10.6026/97320630018780

**Published:** 2022-09-30

**Authors:** Arumugam Padmavathi, Ramasamy Vijayaraghavan, Balu Prakash

**Affiliations:** 1 Department of Microbiology, Nehru Arts & Science College Coimbatore, TN, India; 2 Department of Microbiology, PSG College of Arts and Science, Coimbatore, TN, India; 3 Department of Biotechnology, School of Life Sciences, Vels Institute of Science, Technology and Advanced Studies [VISTAS], Pallavaram, Chennai, Tamil Nadu, India

**Keywords:** Structural characterization, active site prediction, keratinase, Bacillus flexus

## Abstract

Sixteen keratinolytic bacteria were isolated from poultry farm soil samples. The highest keratinlytic enzyme producers of Bacillus flexus was confirmed with 16S rRNA sequence analysis. It is of interest to understand the binding efficiency of the modelled keratinase from Bacillus flexus with different substrates using molecular docking studies. Data provides insights for the identification of substrate recognition patterns, and the development of suitable enzymes to improve their use in keratin degradation.

## Background:

Feathers are considered a by-product of poultry production, the untreated feather waste as source of many pathogenic microorganisms. Poultry feather contains 90% keratin which is highly disulfide-bonded and it's not easily to degradation even treated with various proteases such as pepsin, papain, and trypsin [1]. Despite this, various studies confirm that keratin, which has a hard structure, can be degraded by bacterial keratinase enzyme. Numbers of Bacillus species are characterized as keratinase producers, i.e., B. subtilis, B. cereus, Bacillus licheniformis, B. megaterium, and B. pumilus. Keratinases belong to the subtilisin group, serine protease [2]. It's worth noting that keratinases have a proclivity for green technologies. Decomposing keratin is keratinaseis a green technology with no side effects. Therefore, it is essential to look for novel bacterial strains with keratinolytic activity and to explore their keratinolytic potential from a variety of contexts.Hence, it is interesting to screen, isolate and identify potential keratinolytic bacteria. In addition, the keratinolytic bacteria and their function of keratinasewere identified.The molecular docking method is used to describe the atomic level relationships between a molecule and a protein, which allows to classify molecular behavior at target protein binding sites and to understand important biochemical processes [3]. Therefore, it is interest to understand the binding potential of keratinase enzyme obtained from Bacillus sp.

## Materials and method:

### Isolation of keratinase producing isolates:

About 1 gm of soil was collected from poultry farm and suspended in 9 ml of saline solution (0.9 % NaCl). A volume of 0.1 ml of suspension is serially diluted and then spread on the nutrient agar plate. The plate was incubated at 37°C for 24 hrs. The colonies grown were screened for the keratinase production [4].

### Screening of keratinase producing isolates:

Amongst the different bacterial colonies obtained on the nutrient agar each was inoculated onto a sterile feather meal agar plate. The Feather meal media 1 (g/l): NaCl, 0.5; K2HPO4, 0.3; KH2PO4, 0.4 and Feather, 10; and pH was maintained at 7.5 at 30°C for 7 days, isolates which exhibited the largest clearing zones were selected, identified and grown in cultivation media for enzyme production. The potential isolates were selected based on the isolates showed positive result on the screening test [4].

### Identification of keratinase producing isolate by 16srRNA sequence:

The 16srRNA sequence was carryout in medauxin, Bengaluru, India. The forward and reverse DNA sequencing reaction of PCR amplicon was carried out with forward primer and reverse primers using BDT v3.1 Cycle sequencing kit on ABI 3730xl genetic Analyzer. The 16S rRNA gene sequence was used to carry out BLAST with the database of NCBI Genbank database. Based on maximum identity score first ten sequences were selected and aligned using multiple alignment software program Clustal W. Distance matrix was generated and the phylogenetic tree was constructed by using MEGA 7.

### Docking analysis:

Sequence Selection Method 1: At first, the 16s RNA sequence of keratinase producing Bacillus flexus strain was converted into the corresponding protein sequence using an online Insilico tool, DNA-Protein sequence tool (https://biomodel.uah.es/en/lab/cybertory/analysis/trans.htm).

### Sequence Selection Method 2:

The Feather containing keratin protein sequence of Gallus gallus (KRFC_CHICK: P02450) was retrieved from UniProt (https://www.uniprot.org/) database in FASTA format and secondary structure analysis was done using GOR IV tool (https://npsa-prabi.ibcp.fr/cgi-bin/npsa_automat.pl?page=/NPSA/npsa_gor4.html). Protein motif analysis was also done using Scan ProSite tool (https://prosite.expasy.org/scanprosite/) on Keratin.

### 3D Structure Prediction:

The protein sequence was converted into 3D structure using an automated protein modelling server, SWISS MODEL (https://swissmodel.expasy.org/).

### 3D structure visualization:

The modelled 3D structure of Keratinase obtained from Bacillus flexus strain and the Feather keratin protein of Gallus gallus were visualized using an advanced molecular visualization tool, Discovery Studio in order to perform the molecular drug docking studies.

### Drug docking studies:

The modelled protein targets, Keratinase and (Gallus gallus) were docked by an automated molecular docking server, PatchDock (https://bioinfo3d.cs.tau.ac.il/PatchDock/). Patchdock is a molecular docking algorithm based on geometry. The Atomic Contact Energy (ACE) score predicted by the Patchdock server is used to calculate the change in desolvation energy of the proteins. An ACE value which is negatively high indicates more favourable desolvation-free energy. After this, the docking results were validated using Discovery Studio Software to visualize the H-bond interaction and drug –protein bind affinities [5].

## Results and Discussion:

A total of 28 bacterial isolates were isolated from samples of poultry farm soil, in Namakkal, India. Upon preliminary screening, the 23 isolates showed proteolytic activity, forming a significant hydrolytic zone of clearance around their colonies confirming the degradation and utilization of skim milk. In case of keratinolytic activity, 16 of were showed positive results. According to biggest zone of clearance producing isolates on both media, which isolates (n=2) were selected for Keratinase production as a second selection in the basal mineral media using feather as the sole carbon; isolate 19 showed keratinase activity of 48.7U/mL. Based on the culture character, morphological character and biochemical character, such isolate as Bacillus sp. Among other producers, Bacillus is one of the best keratinase producers, with a higher production capacity of this enzyme. Many studies have reported that Bacillus is the dominant species for keratinase production [6-7]. In 2018, a previous study by Khodayari and Kafilzadeh [4] observed keratinase production of various Bacillus species from soil samples contaminated with poultry soil. The selected keratinolytic bacterium was identified based on the 16S rDNA sequence.

Sequence analysis noted a DNA fragment about 1500 bp confirming the 16S rRNA genes. The 16S rDNA sequence was analysed with blast program in NCBI was identified as Bacillus flexus strain with about 100% homology to available sequences and phylogenetic analysis also utilized for confirmation of the isolate. Similarly Khodayari and Kalzadeh [4] also found similar result of 16srRNA analysis. In this study, next part of the investigation was molecular docking analysis for binding efficiency of the modeled keratinase from Bacillus flexus. At first, the secondary sequence was analysed using GOR 1V [8] tool in order to predict the various structural elements present in the sequence product of 16s RNA (Bacillus flexus) (Figure 1 & Figure 3 - see PDF) clearly represents that Alpha helix (Hh) is 17.42 %, Extended Strand (Ee) is 16.85% and Random Coil (Cc) is 65.73%. The total length of the gene product is 577bp and that of the respective amino acids is 178aa. The keratin protein sequence (Figure 2 - see PDF) was analyzed using ScanProsite tool [9] in order to identify the active sites present. Based on the overall result of protein profiling studies the functional motifs present in Keratin are shown in Table 1(see PDF): these motifs regions play one major role in the post-protein-protein docking. In protein profiling studies, it was found that the amino acids coding our sequence product show the various functional motif regions based on the ScanProsite tool [9]. Among 178 aa length of the protein, the potential motif regions are as follows: Casein kinase II phosphorylation site (PS00006) Phosphosereine : 2-5 (SCFD) , 18-21(SCNE))N-myristoylation site (PS00008) N-myristoylation site : 58-68 (GSSTSA), 66-71 (GSILSE), 73-78 (GVPISS), 87-92 (GSRFSG) Protein kinase C phosphorylation site (PS00005) Phosphosereine : 91-93 (SGR) Amidation (PS00009) Amidation site : 91-94 (SGRR) [10] (Figure 4 - see PDF).

Keratinases are serine and metallo proteases consist of conserved residues which form their active sites. The structural foundation for their activity is revealed by the crystal structures of various keratinases and can be used to produce more stable and effective enzymes for use in industries [11]. Homology models for other keratinases can be generated using these structures [11]. Keratin consists of large amounts of carbon, nitrogen, and sulphur that can be modified into a diversity of natural compounds [12]. As they are capable of breaking down keratins, keratinases have several applications in industries and biotechnological institutes [13]. Our sequence was also based on 3D structure prediction using automated homology modelling server SWISS MODEL [14]. Figure 5(see PDF) represents the various 3D models of the modelled protein. Based on researches, several mechanisms have been suggested [15]. The presence of a high amount of disulfide bonds makes keratin breakdown difficult [16]. Because the majority of keratinases are proteases that break peptide bonds, other enzymes or substances are required to impact the disulfide bonds and decrease the pressures for keratin packing in order to make proteins accessible to the proteases [17]. As a result, keratin degradation involves at least two processes, including disulfide bond breakage and proteolysis. Mechanical destruction, the generation of inorganic sulfite, and the participation of disulphide reductase have all been found to contribute to the phase of disulfide bond breakdown [16]. Keratinases can degrade polypeptides into amino acids during the protein breakdown phase. Keratin breakdown is mediated by numerous protease families [18]. Various mechanisms have been suggested on the basis of studies collected [15-16]. Owing to a large number of disulfide bonds in keratin it becomes difficult to degrade it [16]. Since a large number of keratinases are proteins for breaking peptide bonds, other enzymes or chemicals are required to break the disulfide bonds and decrease the compactness of keratin packing in order to enable proteins to access the proteases [17]. Our modelled feather keratin protein target is chosen as ligand and Keratinaseof Bacillus flexus as a receptor. The docking results of HDock server [19] describe the docking scores based on the intermolecular binding affinities. Figure 7(see PDF) clearly elucidate that the Keratinase of bacillus sp binds keratin protein at various binding sites. There are four potential binding sites in Keratin protein, namely, Casein kinase II phosphorylation site, N-myristoylation site, Protein kinase C phosphorylation site, Amidation site. Interestingly, we observe that keratinase binds at the N-myristoylationsite and phosphorylation site of Keratin at amino acid positions (Figure 8, 9 & 10 - see PDF) (motifs) 21,66 and 73.These various keratin proteins contribute to the h bond formation with most compounds, indicating that these residues might be crucial to enzymatic hydrolysis in the degradation pathway.

## Conclusions:

The overall results obtained from this study elucidate that the keratinase enzyme of Bacillus flexusis is bound to the feather keratin protein of Gallus gallus, which helps in disulphide bond breakdown in keratin.
